# Green tea extract addition into a Tris-based egg yolk extender improves Bali bull sperm quality

**DOI:** 10.5713/ab.22.0184

**Published:** 2022-09-06

**Authors:** Ragil Angga Prastiya, Tri Wahyu Suprayogi, Aldea Erian Debora, Ani Wijayanti, Anny Amalia, Deny Sulistyowati, Aras Prasetiyo Nugroho

**Affiliations:** 1 Veterinary Reproduction Division, School of Health and Life Sciences (SIKIA), Universitas Airlangga, Surabaya 60115, Indonesia; 2 Division of Veterinary Reproduction, Faculty of Veterinary Medicine, Universitas Airlangga, Surabaya 60115, Indonesia; 3 Singosari National Artificial Insemination Center, Directorate General of Livestock and Animal Health, Malang 65153, Indonesia; 4 Animal Husbandry Science Department, Faculty of Animal Husbandry, Jenderal Soedirman University, Purwokerto 53122, Indonesia

**Keywords:** Applied Genetics and Breeding, Bali Bull, Cryopreservation, Extender, Green Tea Extract, Livestock and Gene Bank

## Abstract

**Objective:**

The conservation of Bali bulls, the Indonesian native breed of cattle, is crucial for cattle breeding in Indonesia. To guarantee the spread of Bali bulls through artificial insemination the quality of the frozen semen must be high. To this end, adding an extender material to semen that increases spermatozoa’s survival during cryopreservation is important. Green tea extract (GTE) can be used as cryoprotectant because its high antioxidant activity can help avoid reactive oxygen species formation.

**Methods:**

Semen of five Bali bulls from the National Artificial Insemination Center at Singosari, Indonesia was collected routinely twice a week. First, fresh semen inspection was performed to determine the feasibility of using Bali bulls as animal samples. The extender used in this study was Tris-based egg yolk. The samples were divided into four treatments: T0, no GTE added to the extender; T1, 0.05 mg GTE plus 100 mL extender; T2, 0.10 mg GTE plus 100 mL extender; and T3, 0.15 mg GTE plus 100 mL extender. The semen freezing process was conducted according to standard procedures and sperm quality parameters, i.e., sperm motility, viability, abnormalities, and membrane integrity observed pre-freezing and post-thawing.

**Results:**

There were significant differences in total motility, progressive motility, viability, and integrity membrane of Bali bull sperm at both pre-freezing and post-thawing after adding GTE into the extender. In contrast, there were no differences in abnormalities among treatments.

**Conclusion:**

Adding GTE at a 0.15 mg into 100 mL Tris-based egg yolk extender can improve the quality of cryopreserved Bali bull sperm.

## INTRODUCTION

Bali bulls (*Bos sondaicus*), resulting from *Banteng* (*Bos/Bibos Banteng*) domestication, are the most common native cattle breed in Indonesia. Bali bulls are highly resistant to parasites and can adapt to tropical environments and tropical vegetation [1]. Because of its high population, Bali bulls are the main source of meat for human consumption in Indonesia. In Indonesia, Bali bulls play an important role in cattle breeding both at the Government’s Livestock Breeding Center and in smallholder farming scenarios. The reproductive performance of Bali bulls is assessed through the semen quality [2], which is influenced by several factors, including environmental conditions such as season, humidity, temperature, feed, and genetic factors. For instance, the bull’s age and physical soundness can affect semen production and quality, particularly after puberty. Bali bull, which is one of the male cattle sources for frozen semen production, must have good performance to ensure that the volume and quality of fresh semen collected have met the standards [3].

To achieve the sustainable breeding of Bali bulls, good re productive performance of both female and male cattle is important to achieve genetic improvement, particularly when driven by artificial insemination (AI) programs [4]. AI has been effectively used in livestock species like cattle, sheep, and goats. One of the factors influencing the success of AI is semen cryopreservation. The physical and biochemical properties of semen are affected by freezing. The quality of frozen semen is determined by the type and composition of the extender, semen production, AI, temperature, and storage duration [5]. Post-thawing, semen quality depends on the composition of the cryopreservation medium. Sperm death post-thawing is generally associated with osmotic and membrane changes, and inter- and intracellular ice crystal formation during cryopreservation. As semen cryopreservation can cause sperm damage, an extender material is required to protect sperm from bacteria and cold shock and improve the fertility of frozen semen post-thawing [6]. The extender contains ingredients that maintain osmotic pressure and the electrolyte balance, supply food, and energy sources to buffer and inhibit microbial growth. Moreover, it is necessary to add cryoprotectants to the extender to limit the damaging effects of cryopreservation [7].

There are several semen extenders available, including from animal protein sources such as egg yolk or plant protein sources. In cattle, most extenders contain 20% egg yolk or a similar substitute. Particularly, in bulls egg yolk provides an excellent sperm protection against early cold shock [8]. Recently, the effects of adding a green tea extract (GTE) to the extender used for buck, ram, and boar semen have been reported. Moreover, GTE maintained the semen quality in dogs, pigs, bison, mice, and buffalo. However, only few studies reported on their effect on cryopreservation in bulls [9].

GTE contains numerous polyphenols, especially catechins, with well-confirmed antioxidant properties. GTE is rich in (−)-epigallocatechin-3-gallate (EGCG), which acts directly by inhibiting radical oxygen species (ROS) formation or indirectly by promoting the endogenous defense system [10]. Therefore, the present study aimed to determine the effect of adding GTE to a Tris-based egg yolk extender on Bali bull sperm quality for cryopreservation pre-freezing and post-thawing.

## MATERIALS AND METHODS

### Ethical approval

The experimental procedures were approved and complied with the guidelines of the Animal Ethics Committee of the University. The Research Ethics Committee of the Faculty of Veterinary Medicine, Airlangga University reviewed and approved this study [Approval no.: 1416/UN3].

### Animals

We sampled semen from five Bali bulls aged 5 to 12 years, with body weight ranging between 450 to 600 kg. The animals were clinically declared healthy, had normal genitals and good libido. The bulls were housed in individual cages at 22°C to 27°C. Bali bulls belonged to the National Artificial Insemination Center (NAIC) at Singosari, Malang, East Java, Indonesia.

### Study period and location

This research was conducted at NAIC in Singosari, Malang, East Java, Indonesia. NAIC is a governmental agency under the ministry of agriculture that supplies frozen bull semen for many regions in Indonesia, particularly East Java; recently, it has expanded to overseas markets and ASEAN countries. This study was conducted from December 2021 to February 2022. Semen was collected twice a week in the morning using an Artificial Vagina (AV). Ejaculates were processed into frozen semen in compliance with the guidelines of the Indonesian National Standard SNI: 4869-1:2017 (frozen semen production and analysis) of the National Standardization Agency of Indonesia. GTE was prepared at the UPT Laboratory of Herbal Materia Medica Batu Malang, and the freeze-dry process was performed in the biomolecular laboratory of the Faculty of Veterinary Medicine, Airlangga University.

### Green tea extract preparation

GTE was derived from tea leaves purchased from the Wonosari tea plantation in Malang, Indonesia. Green tea leaves weighed 500 g and were separated from the branches. After drying, they were ground into powder using a grinder and mixer and filtered various times to obtain a fine powder. The powder was weighed and adjusted according to treatment needs. Dried green tea leaves were extracted using the maceration method for 3 days with 96% ethanol solvent and covered with aluminum foil to prevent evaporation and obtain better extraction results. The soaked green tea leaves powder was squeezed and collected using filter paper. Then, the product on the filter was evaporated using a rotary evaporator at 50°C and a speed of 45 rpm to separate the solvent and obtain a thick green tea leaf extract. Once the thick GTE was obtained, it was freeze-dried for 1 week to produce a GTE powder by first grounding over a petri dish, and further sieving. The GTE powder was stored in a sterile place before use [11].

### Semen collection

Semen was collected using an AV made of rubber including a tube, inner liner, binder, funnel, and a collection tube on a Proper sterilization scale. The bulls were stimulated using teasing cows. Water with at 50°C to 55°C was inserted into the AV through the nipple hole until half of it was closed and pumped. Vaseline was applied to the AV canal up to approximately 1/3 to 1/2 of the AVl inner side length. When the Bali bull approached and wanted to ride the teaser female, the male’s prepuce was grasped, and the penis directed into the AV during ejaculation. The AV was removed and either immediately examined in the laboratory or temporarily stored at 5°C (on a thermostat or crushed ice on a towel). The AV was covered with a jacket to protect the spermatozoa from sun exposure. Warm glass bottles were also used for collection. Two consecutive ejaculates were collected at weekly intervals for four weeks. Immediately after ejaculation, the collected semen was transferred to the laboratory and stored in a water bath at 33°C to 34°C. The semen sample was evaluated macroscopically for volume, color, consistency, and pH. A microscopic examination was also conducted to evaluate mass motility, individual motility, and abnormalities. Fresh semen having >80% motility and <15% abnormality qualified for this study [12].

### Extender preparation

Fresh semen meeting the above requirements was further processed to the dilution stage. The semen was diluted using an egg yolk Tris extender containing Tris aminomethane 1.6%, citric acid 0.9%, lactose 1.4%, distilled water 80%, raffinose 2.5%, egg yolk 20%, penicillin 100,000 IU/100 mL, streptomycin 0.1 g/100 mL, and 13% glycerin according to the National Standardization Agency of Indonesia. Raffinose pentahydrate, citric acid, lactose, and Tris aminomethane were homogenized with 80 mL of distilled water using a stirrer for 10 min: then, the temperature was decreased from 100°C to 37°C. At that point, penicillin and streptomycin were added to the solution and homogenized for 10 to 15 min. Up to 20 mL solution was mixed into the egg yolk solution separated from the egg white using filter paper, and subsequently homogenized for 30 min. The extender was stored in the fridge at 4°C for 24 h before use [13].

### Experimental design

This study used a completely randomized experimental design with four treatments and five replicates. The first treatment was a Tris-based egg yolk extender without GTE (T0), the others were a Tris-based egg yolk extender added with GTE of 0.05 mg/100 mL extender (T1), a Tris-based egg yolk extender with GTE of 0.10 mg/100 mL extender (T2), and a Tris-based egg yolk added with GTE of 0.15 mg/100 mL extender (T3). Spermatozoa quality was evaluated both pre-freezing and post-thawing.

### Inclusion level of and equilibration of green tea extracts in the extender

**Figure 1:**
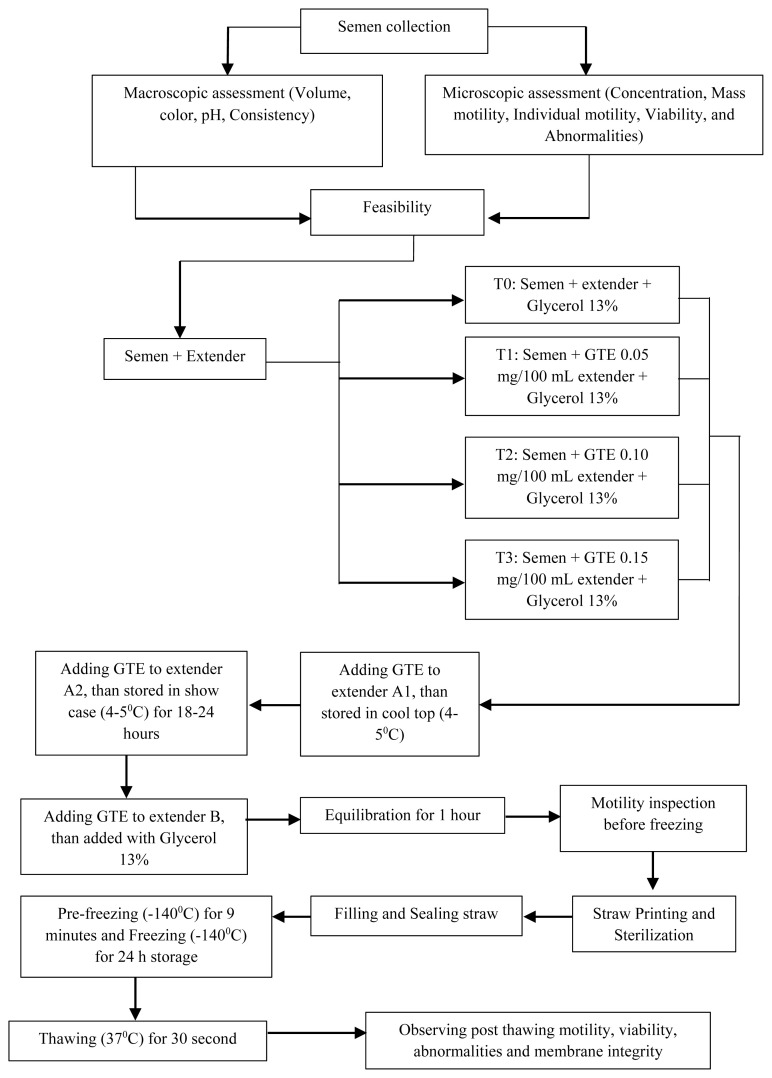
Procedure for adding green tea extract to the Tris-based egg yolk extender.

We prepared three types of extenders, extender solutions A1 and A2 with extenders and GTE, and solution B which contained extenders, GTE, and 13% glycerol. Extender A1 was mixed with semen with a 1:1 ratio. The semen mixed with extender A1 was placed in a Cool-top (4°C to 5°C). The addition of the A2 extender into the A1 extender was performed at the same temperature. The extender B volume was 1/2 of the total volume. The solution was mixed gradually at different volumes according to the predetermined extender formula. Once added to the extender, the semen solution was stored in at 4°C to 5°C and equilibrated for 1 h. Then, pre-freezing and freezing was performed (Figure 1).

### Sperm quality assessment

The evaluation of total and progressive sperm motility was performed with Computer-Assisted Semen Analysis software (Sperm Analyzer IVOS II; Hamilton Thorne, Beverly, MA, USA). Per sample, 10 μL of semen was taken using a micropipette and mixed with 40 μL of saline solution on a slide with a glass cover. Observations with a 200× microscope objective were conducted to determine sperm motility in seven fields with values ranging between 0% and 100%. Sperm concentration measurements were performed using an SDM 6 photometer (Minitube, Tiefenbach, Germany). To that end, 35 μL of fresh semen was placed into a special cuvette filled with 3.5 mL (1:100) saline solution, after homogenization, it was placed on the photometer. Before running the program, according to the manual, the bull identity and sperm motility percentage were entered into the photometer. Sperm viability analysis was performed using the eosin-nigrosine staining method. A total of 5 μL of semen was poured on a slide and 20 μL of eosin-nigrosin was added. The mixture was then homogenized. The mixture was smeared on a slide, dried on a heating table, and observed with a 400× microscope in 10 plane points. Alive sperm have a white head, while dead sperm have a pinkish head. Sperm integrity membrane analysis was carried out through the hypoosmotic swelling solution (HOS) test. A total of 20 μL of semen was mixed into a microtube filled with 1,000 μL of HOS solution, and homogenized. The microtubes were incubated at 37°C for 30 min. A total of 10 μL was transferred onto a slide with a glass cover, then observed with a 400× microscope at 10 points. Sperm with plasma membrane integrity have a circular tail reaction. Examination of spermatozoa abnormalities was conducted using the IVOS device by dripping a diluted semen solution in 0.9% NaCl on an object glass, covered with a cover glass, and then inserted into IVOS for automatic reading. The examination was based on secondary abnormalities (broken, coiled, and folded tail) [14].

### Data analysis

For all parameters, means and standard error of the mean were calculated. One-way analysis of variance with Duncan’s multiple-distance test (SPSS 23.0) was used to detect statistical differences between bulls. The differences were considered significant at p<0.05.

## RESULTS

### Assessment of Bali bull’s fresh semen

The spermatic feasibility of Bali bulls’s fresh semen was evaluated first. Macroscopic and microscopic examinations were performed after semen collection. Table 1 shows the results of the examination of Bali bull’s fresh semen.

### Sperm quality assessment

**Figure 2:**
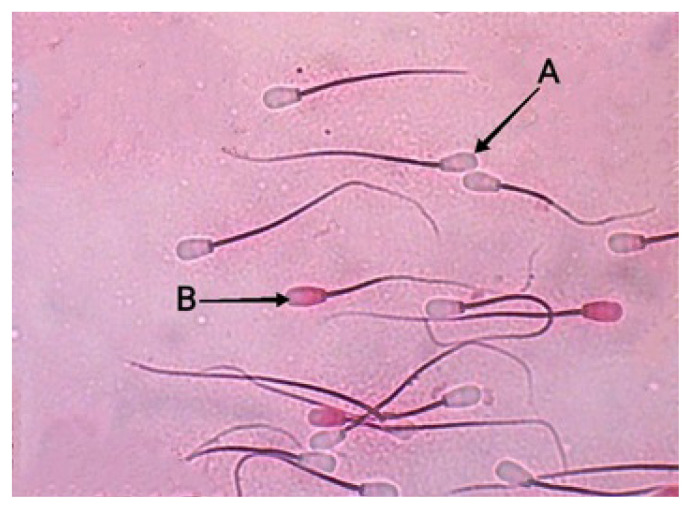
Sperm viability of Bali bulls (*Eosin-nigrosin* staining with 400× microscope magnification). (A) Live spermatozoa are colorless or transparent white. (B) Dead spermatozoa characterized by a colored head.

**Figure 3:**
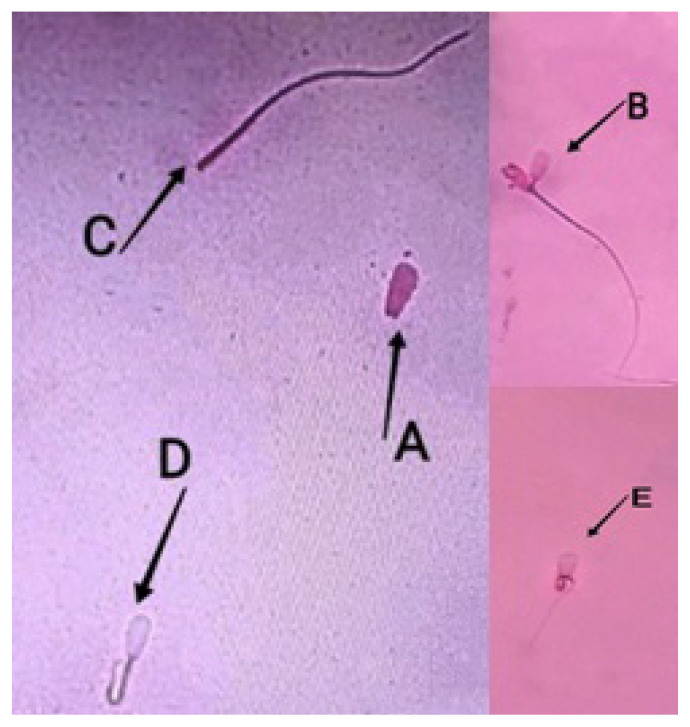
Sperm abnormalities of Bali bulls. (A) No tail, (B) double-headed, (C) broken tail, (D) short tail, (E) coiled tail.

**Figure 4:**
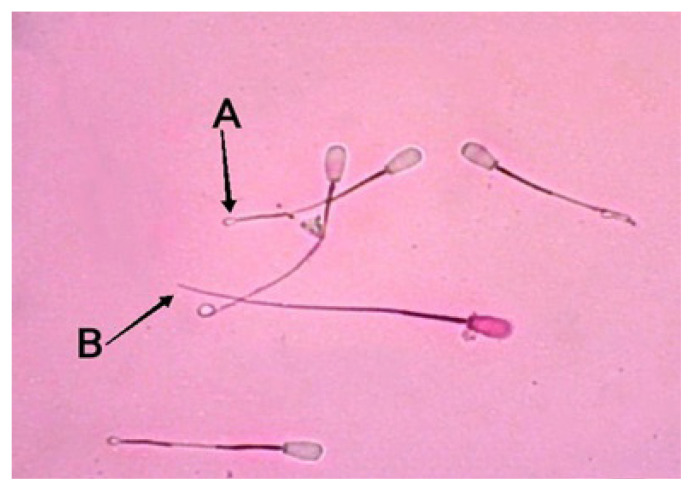
Sperm plasma membrane integrity of Bali bulls as shown by eosin-nigrosin staining under 400× microscope magnification. (A) Spermatozoa with intact plasma membrane and circular tail. (B) Spermatozoa with plasma membrane and straight tail.

Sperm assessment we conducted (viability and abnormalities) using slide preparations stained with eosin nigrosin. Sperm integrity membrane test using HOST. Interpretation of the results in accordance with sperm assessment standards (Figures 2 to 4).

The effect of GTE addition on the progressive motility of spermatozoa during pre-freezing caused significant differences with respect to T0 (p<0.05). Table 2 shows that the addition of GTE at T3 provided the highest progressive sperm motility and was significantly different between T0 and T1. Sperm viability significantly differed from T0with GTE addition at T1, T2, and T3 (p<0.05), and the sperm viability of T3 was significantly different from that of T1 and T2. Adding GTE had a significant effect on the proportion of spermatozoa with intact plasma membrane, with T3 showing the highest membrane integrity (p<0.05). The treatments including GTE did not cause more spermatozoa abnormalities than T0.

Adding GTE into the extender caused significant changes from T0 in total sperm motility and progressive sperm motility during post-thawing (p<0.05; Table 3). All treatments with GTE (T1, T2, and T3) showed total motility and progressive motility >40%, so they were appropriate for AI. T3 yielded the highest sperm total motility and progressive motility compared to T1 and T2. T2 and T3 treatments produced a significantly higher sperm viability (p<0.05) than T0.

## DISCUSSION

The volume of Bali bull semen in this study was approximately 6.28 mL, while the average volume in previous studies ranged between 5.5 to 6.9 mL. The highest ejaculate volume corresponded to 4 and 5 years-old bulls, but the peak volume can be achieved at different ages in various breeds [15]. Bali bull semen volume can vary due to age, climate, temperature, and cage humidity [4]. The color of the semen obtained in this study was milky white, similar to that of normal cattle. In this study, there was no semen reddening or significant discoloration due to penile injuries caused by inflammation or infection. Pink discoloration is caused by the presence of blood from infection in the male reproductive tract, but this was not found in our samples [3]. The semen smell was normal, no foul odor or fishy smell due to infection to the reproductive organs or reproductive tract of the cattle was detected. The pH, measured on a sample of fresh semen, of Bali bull semen ranged between 6.4 to 6.6, and was in the range of normal pH of cattle semen (6.4 to 7.8) [2]. The consistency of the Bali bull semen in this study was thick, and met the SNI standard. Good semen should have a thick consistency [13]. Semen concentration is the number of spermatozoa in one mL of ejaculate, and helps determine the amount of extender to be added to the semen. The concentration of the Bali bull semen in this study ranged between 1,038 to 2,035 million/mL. The percentage of abnormalities in this study was 1.60% to 4.10% with an average of 2.52%±1.08%. From these results, the sperm had an standard level of abnormalities <15%, and was suitable for this research. Based on the percentage of plasma membrane integrity in this study (ranger from 83.30% to 86.30% with an average of 84.32%±1.36%), it was good enough to freeze.

GTE was added to the extender as it can be used by sper matozoa as an additional energy source. In addition, GTE contains vitamins A, B1, B3, B5, C, E, K; vitamins C and K act as antioxidants neutralizing free radicals and protecting cell membrane integrity [16]. Free radicals can reduce the semen quality, particularly sperm motility. Furthermore, GTE also contains vitamin E, which *in vitro* can protect cell membranes against lipid peroxidation [17]. Meanwhile, flavonoids can also act as antioxidants, protecting membrane lipids [18]. This study confirms utility the GTE in maintaining spermatozoa quality, indicated by the plasma membrane integrity obtained at a dose of 0.15 mg in a 100 mL extender. The addition of GTE (Camellia sinensis) did not show a significant effect on the sperm abnormalities of Bali bulls.

Another study showed that GTE contains catechins, epi catechins, gallocatechin, epigallocatechin, gallocatechin gallate, and Epigalocathecin gallate (EGCG). EGCG is the most promising bioactive compound due to its strong antioxidant activity [19], in turn improving sperm motility, and viability, and increase fertility [20,21]. EGCG is a kind of catechin that makes up 13% of the total polyphenols in GTE and can only be found naturally in GTE, while other types of catechins can only be found in fruits and vegetables [22]. The catechin content in GTE can reduce lipid peroxidation and increase antioxidant activity.

The effect of adding GTE to extenders has been examined in several studies. A positive effect of GTE on motility, viability, and membrane integrity was reported with a GTE concentration of 0.75% or 75 μL/mL extender on Achai bulls [23]. The addition of GTE into Tris egg yolk during sperm cryopreservation increased sperm motility and membrane integrity [24,25]. In this study, sperm membrane integrity improved with GTE administration. However, the addition of 50 μM EGCG to an equine extender did not affect sperm quality [24]. This difference may be due to differences in the types of antioxidants and dosage.

GTE was also reported to affect normozoospermic pa tients that is reduced spermatozoa DNA fragmentation when added to the media at a dose of 1.0 ng/mL, but there was no significant difference in ROS levels [24]. GTE indirectly stimulates the endogenous immune system [26,27]. However, further studies are needed to determine the pathway and mechanism of GTE in protecting spermatozoa during cryopreservation [28]. During thawing, sperm experiences rapid temperature changes and extreme osmolarity causing damage to the plasma membrane, including the acrosomal cap membrane. Disintegration of the acrosome and partial removal of the outer acrosome membrane, with acrosome thinning are common changes associated with clotting. This abnormality can be attributed to the formation of ice crystals during extracellular fluid coagulation, causing the sub-acrosomal region to develop [29]. Alternatively, osmotic changes can ruin the structure of lipid membrane structures, causing voltage changes in water channel proteins and ion leakage at the plasma membrane, resulting in morphological changes in cytoplasmic organs and mitochondrial structures [26]. Therefore, thawing at 37°C can maintain osmotic balance, improve lipid configuration, and help maintain the protein’s condition of plasma membrane of sperm. Meanwhile, adding GTE to the extender material can maintain motility, viability, plasma membrane integrity, and sperm DNA integrity [30].

## CONCLUSION

Adding GTE (0.15 mg/100 mL) to an egg yolk Tris extender significantly improved motility, viability, plasma membrane integrity of Bali bull sperm before freezing.

## Figures and Tables

**Table 1:** Assessment of Bali bull’s fresh semen

Fresh semen assessment	Mean				

	Bull 1	Bull 2	Bull 3	Bull 4	Bull 5
Macroscopic					
Volume (mL)	5.8	7.4	6.4	5.0	6.8
Color	White milky	White milky	White milky	White milky	White milky
pH	6.4	6.4	6.4	6.6	6.4
Consistency	Thicky	Thicky	Thicky	Thicky	Thicky
Microscopic					
Mass motility	+++	+++	+++	+++	+++
Sperm concentration (Billion/mL)	1.587	2.035	1.670	1.038	1.903
Sperm progressive Motility (%)	78.7	84.0	79.9	76.8	80.7
Sperm viability (%)	82.7	85.7	84.1	81.1	86.7
Abnormalities (%)	2.0	1.6	1.7	3.2	4.1
Sperm membrane integrity (%)	83.4	85.2	83.4	86.3	83.3

+++, the mass motility of spermatozoa forms large waves and moves fast.

**Table 2:** Effect of green tea extract on sperm quality at pre-freezing

Groups^1)^	N	Sperm concentration (10^6^/dose)	Sperm progressive motility (%)	Sperm viability (%)	Sperm abnormalities (%)	Sperm membrane integrity (%)
T0 (0.00 mg)	5	27.18±0.32	55.32^a^±1.32	69.72^a^±0.20	5.36^a^±1.00	68.76^a^±1.93
T1 (0.05 mg)	5	26.88±0.45	57.60^a^±1.45	76.12^b^±0.44	4.84^a^±1.80	73.12^ab^±1.72
T2 (0.10 mg)	5	27.85±0.21	60.80^ab^±1.76	79.56^b^±0.02	3.54^a^±1.19	78.50^bc^±1.37
T3 (0.15 mg)	5	27.39±0.59	64.50^b^±1.46	83.68^c^±0.29	3.30^a^±1.92	82.86^c^±1.95

Data show all mean±standard error of means (n = 5).

1) T0, without addition of green tea extract; T1, with addition of 0.05 mg green tea extract into 100 mL extender; T2, with addition of 0.10 mg green tea extract into 100 mL extender; T3, with addition of 0.15 mg green tea extract into 100 mL extender.

a–c Means in a column with different superscripts differ significantly at p<0.05.

**Table 3:** Effect of green tea extract on sperm quality post-thawing

Groups^1)^	Sperm total motility (%)	Sperm progressive motility (%)	Sperm viability (%)	Sperm abnormalities (%)
T0 (0.00 mg)	45.19^a^±1.22	38.18^a^±0.94	51.27^a^±0.20	6.17^a^±0.31
T1 (0.05 mg)	50.28^b^±1.08	41.60^b^±0.90	53.44^ab^±0.44	6.02^a^±0.55
T2 (0.10 mg)	50.80^b^±1.76	41.84^b^±0.29	57.86^b^±0.02	4.24^a^±0.76
T3 (0.15 mg)	62.53^c^±1.16	49.22^c^±0.15	61.38^c^±0.29	4.19^a^±0.12

Data show all mean±standard error of means (n = 5).

1) T0, without addition of green tea extract; T1, with addition of 0.05 mg green tea extract into 100 mL extender; T2, with addition of 0.10 mg green tea extract into 100 mL extender; T3, with addition of 0.15 mg green tea extract into 100 mL extender.

a–c Means in a column with different superscripts differ significantly at p<0.05.
